# Estimating Temporal Trend in the Presence of Spatial Complexity: A Bayesian Hierarchical Model for a Wetland Plant Population Undergoing Restoration

**DOI:** 10.1371/journal.pone.0028635

**Published:** 2011-12-06

**Authors:** Thomas J. Rodhouse, Kathryn M. Irvine, Kerri T. Vierling, Lee A. Vierling

**Affiliations:** 1 University of Idaho Department of Fish and Wildlife, Moscow, Idaho, United States of America; 2 Northern Rocky Mountain Science Center, U.S. Geological Survey, Bozeman, Montana, United States of America; 3 University of Idaho Geospatial Laboratory for Environmental Dynamics, Moscow, Idaho, United States of America; National Institute of Water & Atmospheric Research, New Zealand

## Abstract

Monitoring programs that evaluate restoration and inform adaptive management are important for addressing environmental degradation. These efforts may be well served by spatially explicit hierarchical approaches to modeling because of unavoidable spatial structure inherited from past land use patterns and other factors. We developed Bayesian hierarchical models to estimate trends from annual density counts observed in a spatially structured wetland forb (*Camassia quamash* [camas]) population following the cessation of grazing and mowing on the study area, and in a separate reference population of camas. The restoration site was bisected by roads and drainage ditches, resulting in distinct subpopulations (“zones”) with different land use histories. We modeled this spatial structure by fitting zone-specific intercepts and slopes. We allowed spatial covariance parameters in the model to vary by zone, as in stratified kriging, accommodating anisotropy and improving computation and biological interpretation. Trend estimates provided evidence of a positive effect of passive restoration, and the strength of evidence was influenced by the amount of spatial structure in the model. Allowing trends to vary among zones and accounting for topographic heterogeneity increased precision of trend estimates. Accounting for spatial autocorrelation shifted parameter coefficients in ways that varied among zones depending on strength of statistical shrinkage, autocorrelation and topographic heterogeneity—a phenomenon not widely described. Spatially explicit estimates of trend from hierarchical models will generally be more useful to land managers than pooled regional estimates and provide more realistic assessments of uncertainty. The ability to grapple with historical contingency is an appealing benefit of this approach.

## Introduction

Degradation of ecosystems from past land use is a widespread phenomenon and a common target of ecological restoration and adaptive management [1], [2]. A key step in the adaptive management process is monitoring to evaluate progress toward objectives [2], where, for example, trend in a population of an appropriately chosen sensitive species may indicate progress. However, this raises the question of how best to approach the problem of trend detection in such actively managed systems. Managed landscapes often exhibit substantial influence from past land use, and a modeling strategy is required that must be at once flexible and sophisticated enough to handle the multiple sources of variation and uncertainty that arise in such settings. Modeling of trend in environmental monitoring has often been approached with generalized linear mixed models and maximum likelihood or restricted maximum likelihood parameter estimation techniques [3], [4]. However this becomes intractable with complex error structures and “random effects” terms in mixed-models often have no explicit interpretation or meaning, only vaguely informing questions about error-generating processes [5]. Bayesian hierarchical modeling offers a more explicit conceptual and technical framework for tackling many of the complexities likely to be encountered when evaluating trend in managed landscapes, and ecologists are increasingly turning to Bayesian hierarchical approaches [e.g. 5]. Despite the sometimes intractable computational demands, Bayesian hierarchical models generally provide more efficient and realistic accounting of uncertainty in parameter estimates through the specification of error terms at each level of the model, including the covariance parameters themselves [5], [6]. A clear-eyed assessment of uncertainty is a crucial element in the adaptive management process.

Spatial structure is one source of uncertainty that is particularly relevant to modeling population trends in actively managed environments. By definition, these settings inherit past land use patterns which can result in complex spatial structure. Environmental gradients may occur across the study domain, providing an additional exogenous source of spatial complexity. Endogenous sources of spatial structure such as dispersal in the target population may also occur [7] and both sources create a modeling environment rich with spatial information. Under this paradigm, spatial structure is more than just a violation of the independence assumption to be dealt with, but rather becomes a source of important ecological insight that can strongly influence observed trends [8], [9]. Hierarchical modeling offers increased opportunities for harnessing this spatial information that is typically ignored or lost in pooled error estimates obtained from classical single-level models [5], [6], [10], [11].

The results of hierarchical spatial models will therefore be of tremendous interest to land managers and other decision-makers because they can provide explicit estimates of trend from subpopulations such as management units or those defined by other relevant grouping factors. Estimates of trend pooled over multiple subpopulations may be misleading if trends are asynchronous; a pooled estimate can indicate positive trend even while some subpopulations are actually in decline [11]. Spatially heterogeneous land use is an example of how such a pattern might arise. Historical contingency is not easily specified in a single-level model, particularly when the details of past land use are poorly understood. However, because variation in trend caused by past land use is likely to occur in discrete areal blocks (e.g. among old fields), the hierarchical model offers a solution, by way of a grouping factor or stratification within which subsampling occurs.

This approach also establishes a means for dealing with anisotropy (non-stationarity) which also arises in such situations [5], [7], [12]. In a manner akin to stratified kriging [7], spatial covariance parameters can be allowed to vary among subpopulations easily in a Bayesian hierarchical framework. This is an important and accessible improvement over geostatistical models that incorrectly assume an isotropic spatial process, particularly for managed landscapes where boundaries among subpopulations are discrete.

We constructed a suite of Bayesian hierarchical models, each with increasing specification of spatial structure, to evaluate trend from annual samples of density counts in a spatially-structured population of the facultative wetland forb, camas lily (*Camassia quamash* [Pursh] Greene [Agavaceae]). Our study was conducted during 2005–2010, prior to and immediately following the cessation of grazing and mowing on the study area. We also evaluated trend in camas density from a reference site monitored during the same time period. Our restoration study site was bisected by roads and drainage ditches, resulting in distinct subpopulations with different land use histories. We harnessed information from this source of spatial structure by fitting subpopulation-specific intercepts and slopes. We modeled anisotropy with subpopulation-specific spatial covariance parameters. We considered our reference site to be quasi-pristine, having been managed as a protected area for 5 decades. Our objective was to determine if a positive trend in camas density was present following the initiation of passive restoration after accounting for key sources of spatial structure; we hypothesized it would be. Our fully-specified spatial model represents a hierarchical extension of the universal kriging model used in geostatistics to predict values at unobserved spatial locations [6], [10], although our immediate goal was to estimate structural parameters of the model and gain insight into population trend rather than to make predictions per se. Our approach merges two important developments in ecological modeling, geostatistics and Bayesian hierarchical modeling, and represents an application that should translate widely to other actively managed ecosystems with richly structured spatial domains.

## Materials and Methods

### Study system

**Figure 1:**
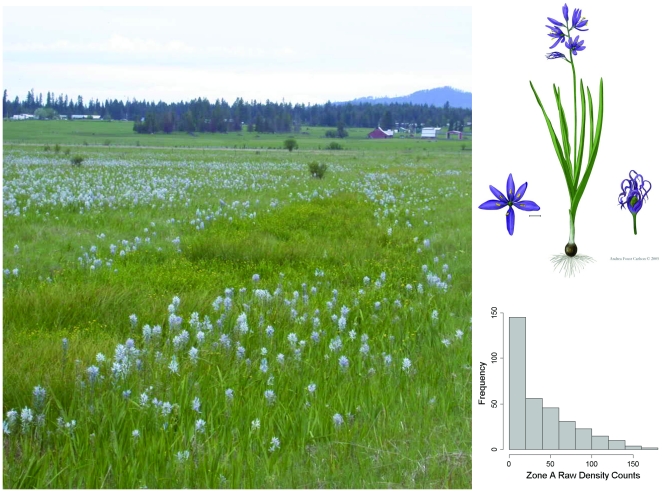
A photograph of camas growing in the restoration study site, Weippe Prairie, Idaho, USA. Camas is a bulb-forming geophyte (upper inset) with limited dispersal capabilities. This attribute and its propensity for prairie swales that experience longer periods of inundation and elevated soil moisture during the growing season creates dense but patchily distributed colonies, as illustrated here. This patchy distribution also leads to highly skewed density counts (lower inset). Upper inset illustration by Andrea Foust Carlson, reproduced with permission. Photo courtesy of the National Park Service.

Camas is a facultative wetland forb species associated with seasonally-inundated wet prairies of northwestern USA and southwestern Canada [13], [14]. It reproduces from large heavy seeds and from bulb offsets that often results in patchily distributed but densely populated colonies (Figure 1). The species was highly prized by indigenous people as a food source [13], [14], [15], and was the focus of major historical events that occurred during harvest that are today commemorated in two US National Park Service units. The extent of these wet prairie ecosystems has been drastically reduced in the region as a result of agricultural conversion and other land use practices [16], [17], [18]. Remaining wet prairies in the region are often structurally altered and compromised by herbaceous non-native and woody native invasive species, and some have been targeted for ecological restoration [e.g. 17,19].

**Figure 2:**
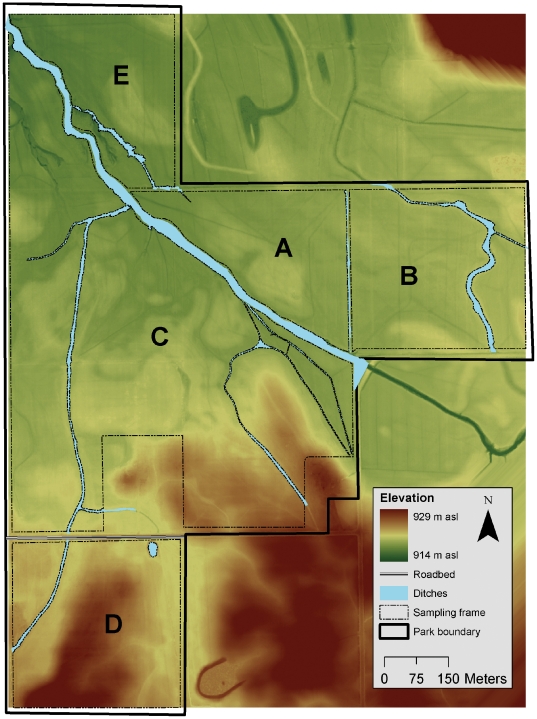
The restoration site, Weippe Prairie, Idaho, USA. The stratified sampling frame identifies 5 zones labeled A–E. A narrow ditch between zone A and B overlaps with the sampling frame boundary and is not readily visible. A paved road separates zone C and D. Elevations were obtained from high-resolution laser altimetry (LiDAR).

In 2005 the National Park Service initiated camas monitoring in a 100-ha portion of the Weippe Prairie (hereafter, the “restoration site”), located in northern Idaho, USA (Figure 2; [20]). The site was acquired from private agriculturalists in 2003 to form a new subunit of Nez Perce National Historical Park. The site had been heavily used as pasture for livestock and some portions were regularly mowed for hay production. Passive restoration began immediately in 2003 with tapered grazing and mowing that was completely discontinued by 2008. Several proposed active restoration strategies were under consideration by park managers, including filling of drainage ditches to restore subsurface hydrology, an important motivation for our study. In 2006, monitoring was also initiated in a 20-ha portion of the Big Hole National Battlefield (hereafter, the “reference site”), located in southwestern Montana, USA [20]. This site has been under National Park Service management since 1963, a 5-decade period of effective conservation protection.

Historic ownership and land use patterns in the restoration site followed the township-range-section pattern established during the US public lands surveys of the 19^th^ century, and are reflected in the contemporary pattern of roads and drainage ditches that partition the site (Figure 2). A straightened and deeply-incised stream channel bisects the study site. These features are substantial and impede surface and subsurface hydrology. Given the dispersal limitations of the species [14], we considered zones to be functionally disconnected by these historic landscape features. The monitoring program was designed to reflect this spatial structure with a stratified sampling frame [20], ensuring that adequate sampling effort occurred in each of 5 recognized subpopulations (hereafter, “zones”; Figure 2). In Figure 2, zones C and D were under different ownership than zones A, B and E prior to NPS acquisition. The intensity and type of land use is believed to have differed among owners, with hay production emphasized over grazing in zone D. Unfortunately, additional details of land use history are lacking for the site, although the condition of zone E was apparently very poor at the time of NPS acquisition and clear evidence of overgrazing in that area persisted until 2008 (Jason Lyon, National Park Service, personal communication).

### Data collection

Each spring camas plants were counted in 4 m×0.15 m (0.6 m^2^) quadrats [20]. A simple random sample of plot locations was drawn for each zone each year. Sample sizes varied considerably during the first 3 years of the study while methods and sampling frame details were refined, and stabilized at 70 plots per zone per year (350 total plots annually) in the restoration site and 150 plots per year in the reference site. Total sample size for the restoration site over the duration of study was 1731 (Text S1); for the reference site it was 682.

In the restoration site we obtained a 1 m resolution (0.064 m vertical accuracy, 0.4 m horizontal accuracy) bare-earth digital elevation model (DEM) produced from low-altitude airborne laser altimetry (LiDAR) collected in 2008 (Figure 2; Terrapoint USA Inc., Woodlands Texas, unpublished report). We used this DEM to measure topographic heterogeneity across the site. We hypothesized a positive association between camas density and the prairie swales that permit prolonged inundation and sustained high soil moisture following snowmelt in the spring. Meso-scale (e.g. 1–10 m) topography is an important source of spatial structure on wetland ecosystems [21], particularly when precipitation and snowmelt controls site hydrology as it appears to do in the restoration site. Acquisition of this high-resolution DEM was critical to enable us to address the question of topographic influences on patterns of camas density. The pattern of ridges and swales across the site forms a maximum topographic relief of only 15 m, and standard 10 m DEMs available from the US Geological Survey are too coarse for use in wet prairies.

### Model building

We hypothesized that cessation of grazing and mowing, a chronic removal of above-ground photosynthesizing tissue of camas plants, would allow surviving plants to replenish carbohydrate stores and allocate more energy into reproduction, yielding an overall positive trend in camas density over time. However, we also expected that trends might vary among zones due to differences in the timing and intensity of historic uses of those zones. For example, zone D was mowed rather than grazed prior to NPS acquisition; such differences might influence contemporary patterns of density (the intercept) and rates of change in density (slope) among zones. Furthermore, given the association of the species with seasonally-inundated wetlands, we expected that prairie topography and the resulting spatial pattern in duration of inundation and soil moisture would also influence patterns of density within zones. Finally, given the limited dispersal capabilities of the species, we expected a strong pattern of residual spatial autocorrelation among observations, even after accounting for the influences of topographic heterogeneity.

**Figure 3:**
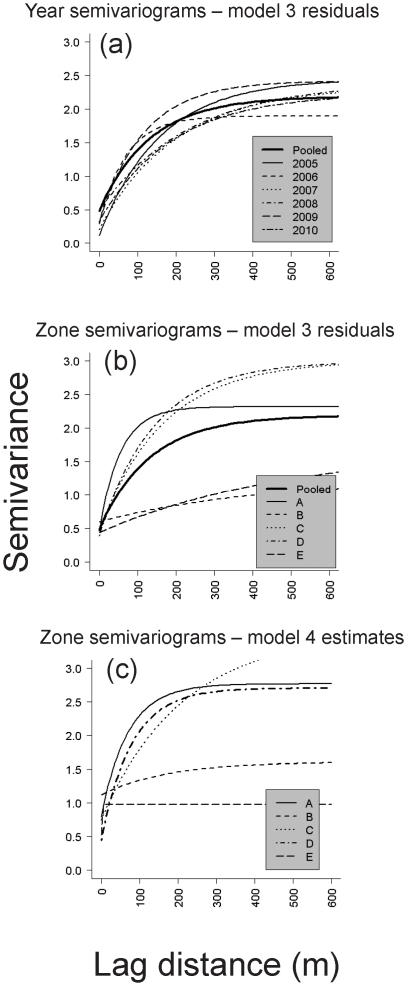
Semivariograms of camas density in the restoration site. In panels a and b, parametric exponential semivariogram models were fit to a) pooled and year-specific and b) pooled and zone-specific empirical models from model 3 residuals for the restoration site. In panel c, zone-specific posterior median estimates for partial sill and range covariance parameters (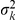, *φ_k_*) obtained from model 4 were used.

We constructed a hierarchical spatial model using a conceptual framework and notational scheme following Wikle [6]. This approach involves the decomposition of a complex joint probability distribution with many parameters into a series of conditional distribution models representing the data-generating process, the latent spatial process, and the parameters. To allow for zonal variation, we indexed the spatial process and the parameter vectors by *k*, representing the zones, an additional level in the hierarchy. Letting 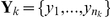
represent camas counts observed in zone *k* over years 2005–2010, our fully spatial model is: 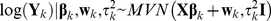
(1)

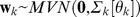
(2)

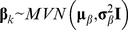
(3)


where **X** = [1,year,elevation], **β** *_k_* = [*β*_intercept,*k*_, *β*_year,*k*_, *β*_elevation,*k*_]^T^, µ*_β_* = [*µ*_intercept_, *µ*_year_, *µ*_elevation_]^T^, 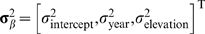
and *θ_k_* = {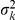, *φ_k_*}, the partial sill and range parameter [6], [10]. We used a lognormal model for **Y** to account for the rather extreme overdispersion observed in the study populations, in which plot counts were distributed with many zeros and very long positive tails of high density counts (Figure 1). We evaluated negative binomial and zero-inflated negative binomial distributional models to describe camas density via goodness-of-fit tests and exploratory models, but found that the high density counts were best described by the lognormal model. We accounted for spatial correlation among plot observations that shared the same (zonal) history by specifying zone-specific slopes and intercepts (equation 3) with common hyperparameters **µ** ***_β_*** and 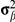 [11]. We assumed an exponential covariance model for 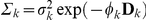, where **D** ***_k_*** is the Euclidean distance matrix for observations in zone *k*, and 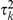
is the nugget of residual variance. We estimated the effective range, **ξ** *_k_* = 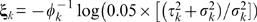
to make inferences about the practical extent of residual spatial autocorrelation [10], [22]. By indexing the spatial covariance parameters by *k*, our model allowed for anisotropy among zones, but enforced stationarity over time, an assumption supported by exploratory semivariograms shown in Figure 3a and 3b.

We chose uniform(0,100) prior distributions for 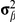 and normal priors on 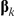 [11]. We used inverse gamma(0.1, 0.1) prior distributions for 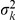 and 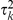 following Banerjee et al. [10]. We used uniform priors for *φ_k_* with informative lower and upper bounds. Following an approach described by Wang and Wall [23], we estimated the lower bound, *φ*_min_ = −log(0.5)×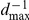, and the upper bound *φ*_max_ = −log(0.01)×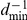, where *d*_max_ and *d*_min_ were the maximum and minimum distances between plot observations. Diffuse priors for *φ* are problematic because it is typically a poorly identified parameter and a variety of approaches to prior specification have been suggested [e.g. 10,23]. The constraints imposed on *φ_k_* allowed for a maximum correlation of 0.5 at the maximum distance between plots, and a minimum correlation of 0.01 at the minimum distance between plots.

In order to better understand how spatial structure influenced the estimation of trend, we also considered three reduced models: a hierarchical model with zone-specific slopes and intercepts but with no assumed residual correlation;



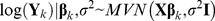
(4)


a partial-spatial hierarchical model as described by equation 4 but where **X** = [1,year] and **β** *_k_* = [*β*_intercept,*k*_, *β*_year,*k*_]^T^, excluding the elevation covariate; and a naive model involving separate regressions (“no pooling” *sensu* [11]) of camas density against year for each zone. For brevity, we refer to these as “model 4”, “model 3”, “model 2”, and “model 1”, respectively. Given our a priori assumption that the full spatial model would provide the most information and the most “honest” accounting of uncertainty, we based all inferences on model 4.

We fit models using OpenBUGS software [24] run from a multi-core processor computing platform with a Linux operating system (Text S2). This provided enough computational speed to obtain a sufficient number (40,000) of MCMC samples from the posterior distribution for model 4 which involved high-dimension covariance matrix decompositions. Following an initial burn-in period of 1000 samples, we thinned the subsequent 40,000 by a factor of 10, which was determined to be adequate from preliminary runs and evaluation of auto-correlation and convergence diagnostics. Inferences therefore were made on posterior distributions from the 3 chains each with 4000 MCMC samples. We evaluated convergence with the Gelman-Rubin diagnostic,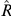; convergence was reached for all parameters according to the criteria 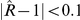 [11]. Bayesian P-values were estimated from the discrepancy in the sum of squared residuals between observed and replicated data (i.e. posterior predictions) as a measure of model fit [11], [25]; P-values ranged from 0.50–0.54 for all models, indicating that the model adequately described the data. Empirical semivariograms shown in Figures 3a and 3b were estimated with the Hawkins-Cressie robust estimator [26]. We centered year and standardized elevation data, which made the intercepts more interpretable and improved parameter estimation and MCMC convergence [11]. We also centered spatial coordinates of the plot locations themselves following Banerjee et al. [10].

## Results

There was clear evidence that camas density patterns varied strongly by zone. Point estimates for zone-specific intercepts, *β*_intercept,*k*_, obtained from our full spatial model (model 4) ranged from −0.06 to 3.02, detailed in Table 1. Because camas density was modeled on the log scale and the year and elevation variables were centered, these intercepts represent zone medians of camas density (per 0.6 m^2^ plot on the log scale) at mean elevation (916.8 m) in the middle of the study period. Zones B and E had the lowest overall density counts throughout the study period, averaging 8 and 5 plants m^−2^, respectively (SDs = 18 and 10 plants m^−2^). Zone C exhibited moderate levels of density, averaging 28 plants m^−2^ (SD = 45), although model 4 intercept variability (SD) was high (Table 1), as a result of a wide range of high and low density patches present in the zone. Zones A and D had the highest observed density counts, averaging 65 and 45 plants m^−2^, respectively (SDs = 65 and 77), with some patches exceeding 200 plants m^−2^. Density estimates in the reference site resembled that of zone E in the restoration site, averaging 5 plants m^−2^ (SD = 12).

**Table 1:** Parameter estimates based on 12,000 MCMC samples from posterior densities obtained from a fully spatial hierarchical model (model 4) fit to camas lily monitoring data collected in Weippe Prairie, Idaho, USA and to a reference site in Big Hole National Battlefield, Montana, USA, during 2005–2010.

	Posterior median	Standard deviation	Lower95% CI^a^	Upper95% CI
*β*_intercept_ Zone A	1.97	0.03	0.48	2.95
*β*_intercept_ Zone B	0.95	0.44	0.14	2.03
*β*_intercept_ Zone C	1.50	0.83	−0.79	3.01
*β*_intercept_ Zone D	3.02	0.74	1.28	4.30
*β*_intercept_ Zone E	−0.06	0.17	−0.42	0.25
*β*_intercept_ Reference	0.55	0.11	0.31	0.76
*β*_elevation_ Zone A	−0.71	0.32	−1.15	0.16
*β*_elevation_ Zone B	−0.81	0.24	−1.34	−0.33
*β*_elevation_ Zone C	−0.81	0.19	−1.22	−0.43
*β*_elevation_ Zone D	−0.75	0.18	−1.07	−0.36
*β*_elevation_ Zone E	−0.85	0.17	−1.22	−0.53
*β*_year_ Zone A	0.10	0.03	0.03	0.17
*β*_year_ Zone B	0.08	0.03	0.01	0.14
*β*_year_ Zone C	0.15	0.03	0.09	0.22
*β*_year_ Zone D	0.04	0.04	−0.05	0.12
*β*_year_ Zone E	0.07	0.03	0.00	0.13
*β*_year_ Reference	0.03	0.02	−0.02	0.07
*µ*_intercept_	1.42	1.13	−0.64	3.73
*µ*_elevation_	−0.79	0.25	−1.19	−0.31
*µ*_year_	0.09	0.05	−0.01	0.18
*ξ* Zone A	185	252	82	881
*ξ* Zone B	285	711	64	2840
*ξ* Zone C	578	715	249	3090
*ξ* Zone D	226	305	113	1030
*ξ* Zone E	0.64	2	0	7
*ξ* Reference	78	22	53	138

a Credible intervals.

Model intercepts (*β*_intercept_) provide log scale estimates of median camas density at mean elevation and year for each restoration site zone and for the reference site. Note that no elevation parameter was included in the reference site model. Model slope parameters (*β*_elevation_, *β*_year_) provide estimates of camas density trend across the low-relief elevational gradient of the restoration site and across time. Hyperparameters (e.g., *µ*_intercept_) used in the zone-specific hierarchical construction of model 4 for the restoration site provide overall site mean estimates of intercept and trend. The effective range (*ξ*) is the lag distance where residual correlation among plots is ≤5%.

**Figure 4:**
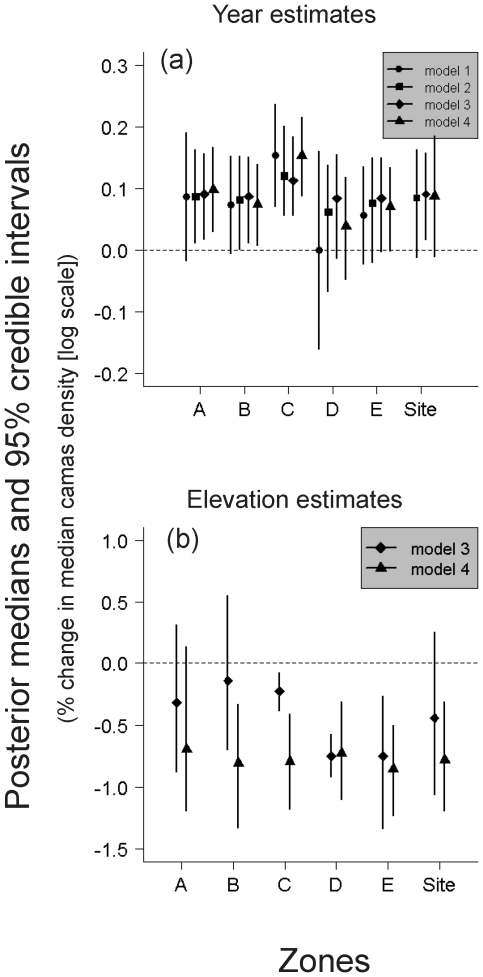
Posterior medians and 95% Bayesian credible intervals for a) trend and b) elevation parameters. Results are for the restoration study site from a series of 4 models of camas density each with successive amounts of spatial structure included. Model 1 represents a spatially “naïve” approach with estimates obtained from independent simple regression models for each zone. Note that no overall restoration site estimate (i.e. *µ*_year_) is generated from model 1. Model 2 is a hierarchical model that allows for zone-specific slopes and intercepts as well as an estimate of *µ*_year_ for the restoration site (labeled “site”). Model 3 is an extension of model 2 with elevation included. Model 4 adds further spatial structure by including zone-specific exponential spatial covariance models that account for residual spatial autocorrelation.

Trend estimates obtained from model 4 were positive for each zone in the restoration site as well as for the site overall, suggesting that passive restoration may be having a desired effect on the camas population (Figure 4a). However, there was considerable variation in the strength of evidence for trend among zones. Zone C exhibited the strongest trend over the 6-year period, with an estimated 16% (*e* ^0.15^) average annual rate of increase in median camas density, and a 95% credible interval (CI) around that point estimate of 0.09–0.22 (Table 1). However, the CI for zones A and B were also >0. There was evidence for a weak trend in Zone E with the lower CI = 0 (Fig. 4a). There was no clear evidence for trend in zone D (95% CI: −0.05–0.12) nor in the reference site during the same time period (95% CI: −0.02–0.07%; Table 1). The SD was consistently low (≈0.3) for trend parameters in all zones (Table 1), even lower for the reference site (0.2), and convergence was quickly reached in MCMC chains, adding confidence to our trend evaluation. 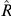 = 1.0 for all 5 zone trend parameters, as well as for the reference site trend parameter.

Topographic heterogeneity appeared to strongly influence patterns of camas density, particularly for zones C, D and E, which had the highest topographic relief on the site (Figure 2). The CI width for elevation was narrower for these three zones than for zones A and B (Figure 4b). As expected, camas density was negatively associated with elevation, and median camas density was estimated to decrease by ≈50–57% (exponentiated), depending on the zone, for a 1 SD (≈1 m) increase in elevation above the mean (Table 1; Figure 4b).

Estimates for the effective range (ξ) and partial sill (σ^2^) both varied among zones (Table 1; Figure 3c), supporting our expectations formed during exploratory analysis (Fig. 3a and 3b) for anisotropy and the need for separate covariance models for each zone. Semivariograms in Figure 3 illustrated that Zones A, C and D had the strongest residual spatial autocorrelation, which was much better described by an exponentially-decaying spatial covariance model than for zones B and E. The lower 2.5% posterior credible intervals for ξ from zones A, C and D ranged from 82–250 m (Table 1), providing evidence for dispersal-driven patchiness at a scale of 10's of meters, despite the high uncertainty in the exact location of those effective ranges (Table 1). Estimated correlation at 10 m lag distance was ≥64% for zones A, C and D, but only 34% for zone B, and <0.01% for zone E. Similarly, median ξ for the reference site was 78 m (Table 1), estimated with relatively high precision (95% CI 53–138 m), and a correlation of 40% at 10 m lag. The CIs for ξ*_k_* in the restoration site were generally wide, but narrowest for zones A and D. Results for zone E indicated a complete absence of residual spatial autocorrelation (Table 1; Figure 3c).

Adding increasing amounts of spatial structure to the model, as represented by a progression from model 1 to model 4, influenced CI width and also adjusted the point estimates of trend (Figure 4a). Statistical “shrinkage” of trend estimates toward *µ*_year_ occurred, particularly in models 2 and 3, with a slight adjustment away from *µ*_year_ after accounting for residual correlation in model 4. A substantial increase in precision of trend (i.e. narrower CIs) was observed with models 2 and 3 after accounting for the exogenous sources of spatial structure, zonal correlation and topographic heterogeneity.

Finally, we note that topographic heterogeneity was very influential to both the point estimates and precision of trend in all zones, and was apparently sensitive to the meso-scale (e.g. 10–100 m) dispersal-related spatial autocorrelation in complex ways. A shrinkage effect stronger than that observed with trend occurred for elevation parameters in model 4, which shifted away from 0 toward *µ*_elevation_ (Figure 4b). There was a complex shift in precision among the elevation parameters as well, where in zones A, C and D, strong residual autocorrelation was apparent and the precision of the elevation parameter decreased in model 4 (Figure 4b). The opposite pattern was observed in zones B and E, where residual autocorrelation was weak.

## Discussion

We developed a spatially-explicit hierarchical model to evaluate trend in the colonial geophyte, camas, following cessation of grazing and mowing in a wet prairie ecosystem. We also applied a simpler spatial model to estimate trend from a reference site for comparison. We found evidence for positive trend following passive restoration. Accounting for spatial structure made a substantial change in our evaluation of trend, however. Results from model 1, a set of independent spatially-naive models for each zone, resulted in rather equivocal evidence for trend; all zones except C included 0 in posterior densities (Figure 4a). Evidence strengthened as we incorporated progressively greater amounts of spatial structure. Results from model 4, our fully spatial model, provided compelling evidence for positive trend over the 6 years of study in 3 zones, and weak evidence in a 4^th^ zone. By comparison, we found no evidence for trend in the reference site over the same period of time, further supporting our conclusion that passive restoration seems to be having a desired effect in at least some portions of the restoration site.

The importance of accounting for underlying differences in zone-specific camas subpopulations was clearly evident. Intercepts, trend, elevation, and spatial covariance parameters all varied in complex ways among zones. High density zones with large intercepts appeared to have the strongest pattern of residual spatial autocorrelation. Zone E, an area that we believe was most heavily grazed prior to and during the first 3 years of monitoring, had very low camas density and no residual autocorrelation. In Zone B, another portion of the restoration site that appeared to exhibit lower than expected density, residual autocorrelation was only weakly evident (Figure 3c). In contrast, the reference site, which had a similar level of density to zones B and E, exhibited the most precise signal for spatial autocorrelation out to a lag distance of ≈80 m. This is interesting because it suggests that colony development and the resulting exponential covariance pattern may strengthen over time in the absence of chronic disturbance. Based on considering residual correlation at 10 m lag and the lower CI boundaries for the effective ranges across zones and in the reference site, a distinct pattern of dispersal-driven patchiness may be typical for our two study populations out to several decameters, but may weaken and become more variable under chronic disturbance and degradation. After accounting for topographic heterogeneity, the two low-density zones believed to be most altered by past land use exhibited the most homogeneity in patch structure. Camas, where present, typically occurred as single plants loosely assembled in undefined patches. This suggests that colony formation might become more distinct over time, reflected in increasing precision of covariance parameters.

The shifts in both the location and scale of trend posterior distributions were noteworthy. Accounting for exogenous spatial structure, i.e. zonal differences and topographic heterogeneity, resulted in increased precision in trend as variation was “mopped up”. Shrinkage effects resulting from the hierarchical construction of the model involving the use of hyperparameters for trend pulled posterior densities closer together and generally away from 0. This makes sense, even for zones with weak effect sizes – in the absence of strong information in one direction or the other, *µ*_year_ provides the best expectation for trend [11].

Less intuitive shifts in location and scale occurred with the elevation parameter that appeared to reflect a complex interaction with residual spatial autocorrelation. In model 4, there was a strong pattern of shrinkage away from 0 toward *µ*_elevation_. This shrinkage effect was not seen in model 3, perhaps due to confounding effects of spatial autocorrelation. The scales at which the spatial processes of topographic heterogeneity and dispersal-driven autocorrelation occur in the restoration site are at distances of tens of meters and therefore overlap. Furthermore, dispersal likely follows the swales with elevated soil moisture in such a way that patch boundaries reflect the underlying topography (Figure 1). This is the likely cause for the divergence in CI widths among zone elevation posteriors seen in Figure 4b. Precision in the elevation CI increased from model 3 to model 4 in zones B and E, the two zones with weak spatial autocorrelation. Precision decreased predictably for zones C and D, and to a lesser extent for zone A, all 3 of which had much stronger residual autocorrelation.

The phenomenon of coefficient shift in regression models when spatial autocorrelation is present yet not correctly modeled has been a topic of recent debate [e.g. 27,28]. Conflicting reports have been made concerning the predictability of shifts in both location and scale in the covariates. In general it is well established that spatial structure in both predictor and response variables result in inflated Type I error [28], [29], [30], as was observed in our inflated precision of the elevation parameter for zones C and D, corrected in model 4. A similar correction was observed in zone D trend, as well as in *µ*_year_. What was striking about our results, however, was that variation in those shifts among zones depended on the strength of autocorrelation and amount of topographic heterogeneity. Furthermore, the shrinkage effect induced from “partial pooling” of slopes and intercepts apparently can override expected coefficient shifts. Beale et al. [28] observed that covariate effect sizes are typically smaller in spatial regression models when residual spatial autocorrelation is present, particularly when true effect sizes are near 0. They suggested this is because non-spatial regression yields less precise estimates, allowing the magnitude of estimates from structurally incorrect models to vary widely. However, our results demonstrate another scenario: in hierarchical models that index covariates by grouping factors, parameters for groups with small effect sizes will shift toward the common mean rather than 0. Groups with small sample sizes will also shift toward the mean because they have less “information” to provide [11]. Modeling spatially-correlated errors therefore can result in a strengthening of that shift, as was demonstrated in Figure 4b, by lowering the effective sample size in groups with strong autocorrelation. We have not found this phenomenon described elsewhere and it was not clear from our study whether this result can be anticipated generally, but it is a scenario likely to be encountered more frequently as the use of hierarchical models increases among ecologists.

Our fully spatial model is a hierarchical extension of the geostatistical kriging models commonly employed for spatial prediction of natural resources [e.g. 10]. Isaaks and Srivastava [12] differentiated between geometric anisotropy, where ranges differ but sills are the same, and zonal anisotropy, where ranges are the same but sills differ, with each type requiring a different strategy to recover the necessary assumption of stationarity [7]. We encountered both types in our study and the flexibility of the Bayesian hierarchical approach allowed us to specify independent covariance models for each zone to accommodate this complex anisotropy. Our strategy is akin to stratified kriging used to interpolate across distinct soil types [31] and forest stands [32]. In situations where anisotropy is not so discrete, this approach may not be appropriate, but we envision many situations where disjunct “steps” in residual covariance patterns are likely, particularly in landscapes modified by human agriculture and roads. Explicitly modeling discrete patterns of spatial covariance improves interpretation and provides insight into spatial processes otherwise masked by globally isotropic models, as we have demonstrated. Furthermore, our approach reduced the *n*-dimensional covariance matrix to smaller *n_k_*-dimensional matrices, a useful computational strategy when estimating spatial covariance parameters by way of complex matrix inversions in geostatistical models (i.e. the “big *n* problem”, [10]).

### Synthesis and applications

Although the importance of spatial structure as both a source of error in model-based inference and as a source of important ecological insight is widely appreciated among ecologists [7], our perception is that it has not been a common topic in restoration and monitoring contexts. Yet we expect that spatial hierarchical models can be widely implemented and useful to practitioners engaged in restoration and adaptive management, in large part because of the coherent integration of large amounts of spatially-explicit information into a single model. For example, we are now able to report on trend following restoration in 5 distinct subpopulations simultaneously, with greater precision compared to the naïve approach represented by model 1. Moreover, our novel insights into camas patch structure can be immediately applied to upcoming restoration decisions. Transplanting of camas bulbs into low swales where no camas colonies are present might be an effective strategy to accelerate recovery, given the species' low dispersal capacity and colonial patch formation. Areas of lower than expected camas density that are impacted by altered surface flow patterns could be targeted for active measures such as ditch plugging and our model can be used to reinforce whether and where such actions are likely to be successful.

The ability to grapple with past land use is a particularly appealing benefit of the hierarchical approach to modeling trend following restoration. Even when the details of site history are vague, the hierarchical model enables this excess variation to be managed more efficiently. For example, Thogmartin et al. [33] specified an additional error term in a Bayesian hierarchical model to account for differences among sites in a restoration context, without attempting to specify the source of the site variation. It was not entirely clear from our observational study whether past land use was in fact driving the differences in trend among zones in the restoration site. Regardless, a considerable amount of correlation in model errors was structured by zone, masking the trend, until it was accounted for with zone-specific parameters.

We achieved additional efficiency and a considerable amount of flexibility by extending our hierarchical construction to the spatial covariance model. The Bayesian approach to inference provided us with an estimate of uncertainty in spatial covariance parameters, by zone, that is not available using other parameter estimation methods [22], [34]. This is an important consideration if a biological interpretation of the modeled endogenous spatial process is desired. By merging Bayesian hierarchical modeling with geostatistics, we have demonstrated one way that long-term monitoring can be a more effective and flexible tool for evaluating actively managed settings. Palmer et al. [35], among others, have called for greater commitment to post-restoration monitoring as a means to not only improve the practice of restoration but to take advantage of the tremendous opportunities that exist for ecological learning that come with monitoring. We hope our study will serve as a motivating example for others to follow in this endeavor.

## Supporting Information

Text S1 Supporting text
(TXT)

Text S2 Supporting text
(DOC)
