# Supplementary material for: Estimating Temporal Trend in the Presence of Spatial Complexity: A Bayesian Hierarchical Model for a Wetland Plant Population Undergoing Restoration
Source: PLoS One. 2011 Dec 6;6(12):e28635. doi: 10.1371/journal.pone.0028635 (PMC3232238; doi:10.1371/journal.pone.0028635)
Supplement: Text S2 — Supporting text (DOC) [file pone.0028635.s002.doc]

**Text S2. BUGS model specification for the anisotropic hierarchical spatial model (model 4) fitted to camas density observations (*n*=1731) made during 2005-2010 in the Weippe Prairie restoration site as described in the article by Rodhouse et al. entitled “Estimating temporal trend in the presence of spatial complexity: a Bayesian hierarchical model for a wetland plant population undergoing restoration”. Data are supplied in Text S1. Note that this model runs slowly with the accompanying (or any large) dataset, start with few MCMC iterations.**

model{

#Priors

for(i in 1:5){

alpha[i]~dnorm(mu.int,int.prec)

beta.year[i]~dnorm(mu.year,year.prec)

beta.elev[i]~dnorm(mu.elev,elev.prec)

spat.prec[i] ~ dgamma(0.1,0.1)

sigmasq[i]<-1/spat.prec[i] #this is the partial sill

error.prec[i]~dgamma(0.1,0.1)

tausq[i]<-1/error.prec[i] #this is the nugget

phi[i]~dunif(0.00056,6.14)

#These bounds determined as described in the text following Wang #and Wall. Phi max comes from zone C, which had the greatest #max(dist). Phi min was similar across zones. Therefore a single #range was suitable across all zones.

}

mu.int~dnorm(0,0.001)

int.prec<-1/(sigma.int*sigma.int)

sigma.int~dunif(0,100)

mu.year~dnorm(0,0.001)

year.prec<-1/(sigma.year*sigma.year)

sigma.year~dunif(0,100)

mu.elev~dnorm(0,0.001)

elev.prec<-1/(sigma.elev*sigma.elev)

sigma.elev~dunif(0,100)

#Likelihood

for(i in 1:n){

y[i]~dnorm(mu[i],error.prec[zone[i]])

mu[i]<-alpha[zone[i]]+beta.year[zone[i]]*year[i]+

beta.elev[zone[i]]*elev[i]+W[i]

muW[i]<-0.0

#Note this hierarchically-centered parameterization follows #Banerjee et al. 2004

}

#Indexing of W by zone – the “stratified kriging” approach

W[1:332] ~ spatial.exp(muW[1:332],UTMX[1:332],UTMY[1:332],spat.prec[1],phi[1],1)

W[333:691] ~ spatial.exp(muW[333:691],UTMX[333:691],UTMY[333:691],spat.prec[2],phi[2],1)

W[692:1172] ~ spatial.exp(muW[692:1172],UTMX[692:1172],UTMY[692:1172],spat.prec[3],phi[3],1)

W[1173:1442] ~ spatial.exp(muW[1173:1442],UTMX[1173:1442],UTMY[1173:1442],spat.prec[4],phi[4],1)

W[1443:1731] ~ spatial.exp(muW[1443:1731],UTMX[1443:1731],UTMY[1443:1731],spat.prec[5],phi[5],1)

#Derived parameters

for (i in 1:5){

range[i]<-1/phi[i]

eps[i]<--range[i]*log(0.05*((tausq[i]+sigmasq[i])/sigmasq[i]))

ratio[i]<-tausq[i]/(sigmasq[i]+tausq[i])

prod[i]<-sigmasq[i]*phi[i]

}

#Assess model fit using sum of squared residuals discrepancy

for (i in 1:n){

residual[i]<-y[i]-mu[i] #residuals

predicted[i]<-mu[i] #predicted values

sq[i]<-pow(residual[i],2) #squared residuals for observed data

#Generate replicate data for posterior predictive checks

y.new[i]~dnorm(mu[i],error.prec[zone[i]]) #one new dataset at #each MCMC iteration

sq.new[i]<-pow(y.new[i]-predicted[i],2) #squared residuals for #new data

}

fit<-sum(sq[])

fit.new<-sum(sq.new[])

test<-step(fit.new-fit)

bpvalue<-mean(test)

} #end script
